# Effectiveness of a critical reflection competency program for clinical nurse educators: a pilot study

**DOI:** 10.1186/s12912-023-01236-6

**Published:** 2023-03-16

**Authors:** Sujin Shin, Inyoung Lee, Jeonghyun Kim, Eunyoung Oh, Eunmin Hong

**Affiliations:** 1 College of Nursing, Ewha Womans University, 52 Ewhayeodae-gil, Seodaemun-gu, 03760 Seoul, Republic of Korea; 2 Department of Nursing, Dongnam Health University, 50, Cheoncheon-ro 74beon-gil, Jangan-gu, Suwon-si, Gyeonggi-do Republic of Korea; 3 College of Nursing, Catholic University of Pusan, 57 Oryundae-ro, Geumjeong-gu, 46252 Busan, Republic of Korea; 4 Department of Nursing, Nursing Administration Education Team Leader, Catholic University of Korea Bucheon ST. Mary’s Hospital, 327, Sosa-ro, 14647 Bucheon-si, Gyeonggi-do Republic of Korea

**Keywords:** Education professional, Nurses, Nursing education research, Program evaluation

## Abstract

**Background:**

Critical reflection is an effective learning strategy that enhances clinical nurses’ reflective practice and professionalism. Therefore, training programs for nurse educators should be implemented so that critical reflection can be applied to nursing education. This study aimed to investigate the effects of a critical reflection competency program for clinical nurse educators on improving critical thinking disposition, nursing reflection competency, and teaching efficacy.

**Methods:**

A pilot study was conducted using a pre- and post-test control-group design. Participants were clinical nurse educators recruited using a convenience sampling method. The program was conducted once a week for 90 min, with a total of four sessions. The effectiveness of the developed program was verified by analyzing pre- and post-test results of 26 participants in the intervention group and 27 participants in the control group, respectively. The chi-square test, independent t-test, Mann-Whitney U test, and analysis of covariance with age as a covariate were conducted.

**Results:**

The critical thinking disposition and teaching efficacy of the intervention group improved after the program, and the differences between the control and intervention groups were statistically significant (F = 14.751, *p* < 0.001; F = 11.047, *p* < 0.001). There was no significant difference in the change in nursing reflection competency between the two groups (F = 2.674, *p* = 0.108).

**Conclusion:**

The critical reflection competency program was effective in improving the critical thinking disposition and teaching efficacy of nurse educators. Therefore, it is necessary to implement the developed program for nurse educators to effectively utilize critical reflection in nursing education.

## Background

The critical thinking of clinical nurses is essential for identifying the needs of patients and providing safe care through prompt and accurate judgment [1–3]. Critical thinking can be practiced through critical reflection [4], a dynamic process in which nurses reflect on their nursing behavior to improve their perspective on a situation and change future nursing practices in a desirable direction [5]. Through critical reflection, nurses grasp the contextual meaning of a situation and reconstruct their experiences to apply their learning in practice, thereby identifying the meaning of nursing [3]. In other words, critical reflection can help nurses convert their experiences into practical knowledge [6]. Thus, critical reflection may be an effective learning strategy linking theory and practice in clinical nursing education [7].

Studies have reported that critical reflection is effective in improving nurses’ reflective practices and professionalism. Teaching methods that use critical reflection can improve nurses’ knowledge, communication skills, and critical thinking abilities [1, 8, 9]. These methods can be effective in improving clinical judgment and problem-solving abilities by providing new nurses with opportunities to apply their theoretical knowledge in clinical practice [10, 11]. In addition, critical reflection has positive effects on the professionalism of new graduate nurses and reduces reality shock during the transition from university to clinical practice [12]. These advantages have led to the increasing application of critical reflection in training programs for new graduate nurses, including nursing residency programs [13–15].

In order to facilitate new nurses’ reflective thinking and practice by clinical nurse educators, educators must be trained to strengthen their critical thinking disposition, nursing reflection, and teaching efficacy competency. Educators help new nurses adapt and develop their expertise in clinical settings [16, 17]. Moreover, continuing education for nurses to improve their teaching competency relates to the satisfaction of learners and nurse educators, which improves the quality of clinical nursing education [18]. Therefore, opportunities for nurse educators to develop teaching competency for critical reflection in education should be provided [19] and educational support for nurse educators to improve critical reflection competency is needed.

However, although there have been studies that have evaluated the effectiveness of the educational interventions concerning critical reflection to new nurses, few studies have been conducted on educational interventions on the critical reflection competencies of clinical nurse educators in charge of educating new nurses. Therefore, this study aimed to investigate the effects of a critical reflection competency program for clinical nurse educators on improving critical thinking disposition, nursing reflection competency, and teaching efficacy.

## Methods

### Study design

**Fig. 1:**
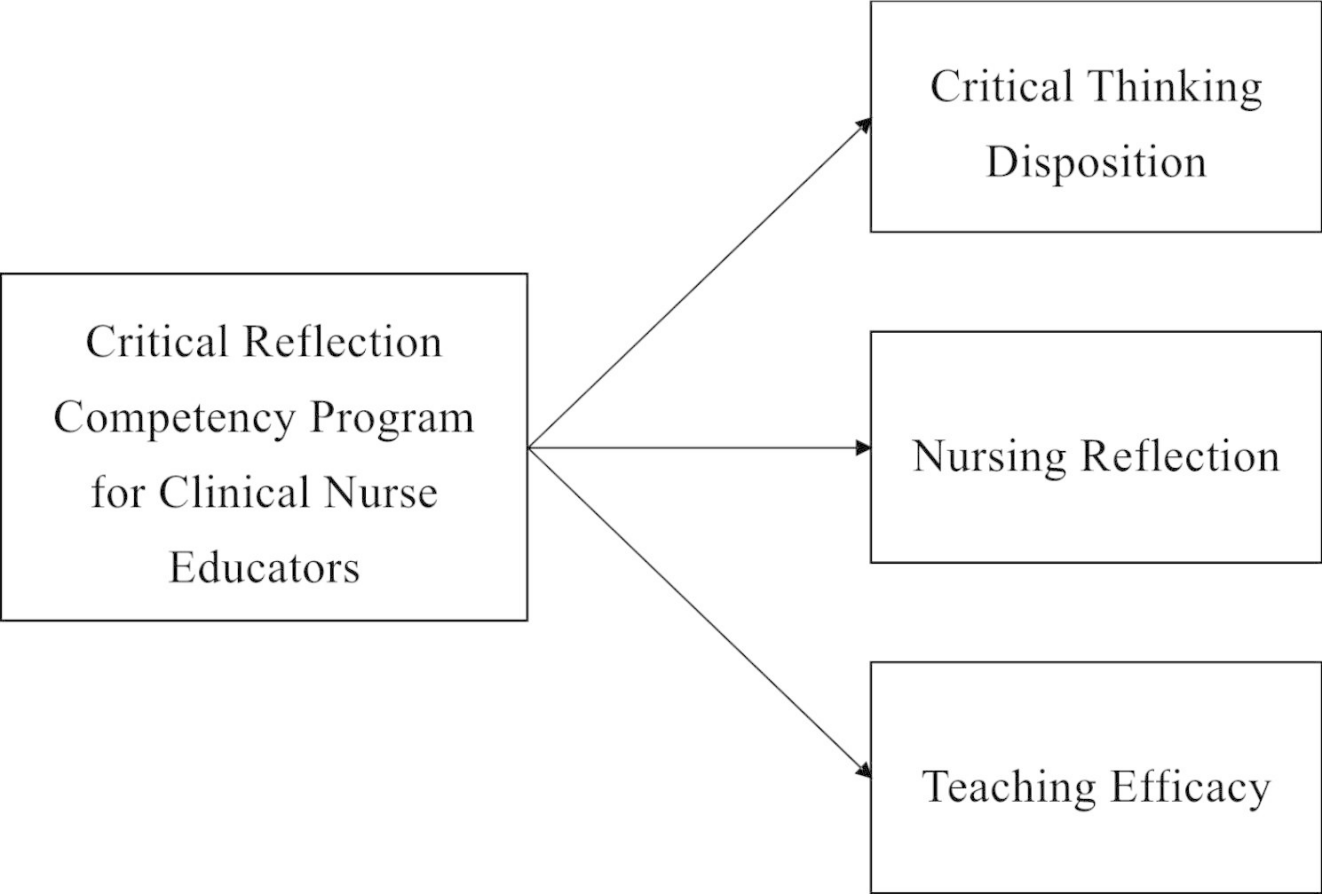
Conceptual Framework

A pilot study was conducted with a pre- and post-test control group design to investigate the effects of the critical reflection competency program on the critical thinking disposition, nursing reflection competency, and teaching efficacy of nurse educators. The conceptual framework in this study was proposed that the critical reflection competency program will improve critical thinking disposition, nursing reflection, and teaching efficacy of clinical nurse educators [Fig. 1].

### Sample

Participants were clinical nurse educators in hospitals who were recruited using a convenience sampling method. Nurse educators were eligible to participate if they had dedicated nursing education in a clinical setting. They dedicated to nursing education focused on staff development of current nurses, especially the education for new nurses. They also included those who completed all four sessions of the program and participated in the data collection before and after the program. A recruitment document was sent to hospitals to recruit the participants, hospitals were selected with concerning role of clinical nurse educators. Participants were recruited from two hospitals of different sizes and the number of participants differed for each hospital, and they were allocated according to the order of registration.

**Fig. 2:**
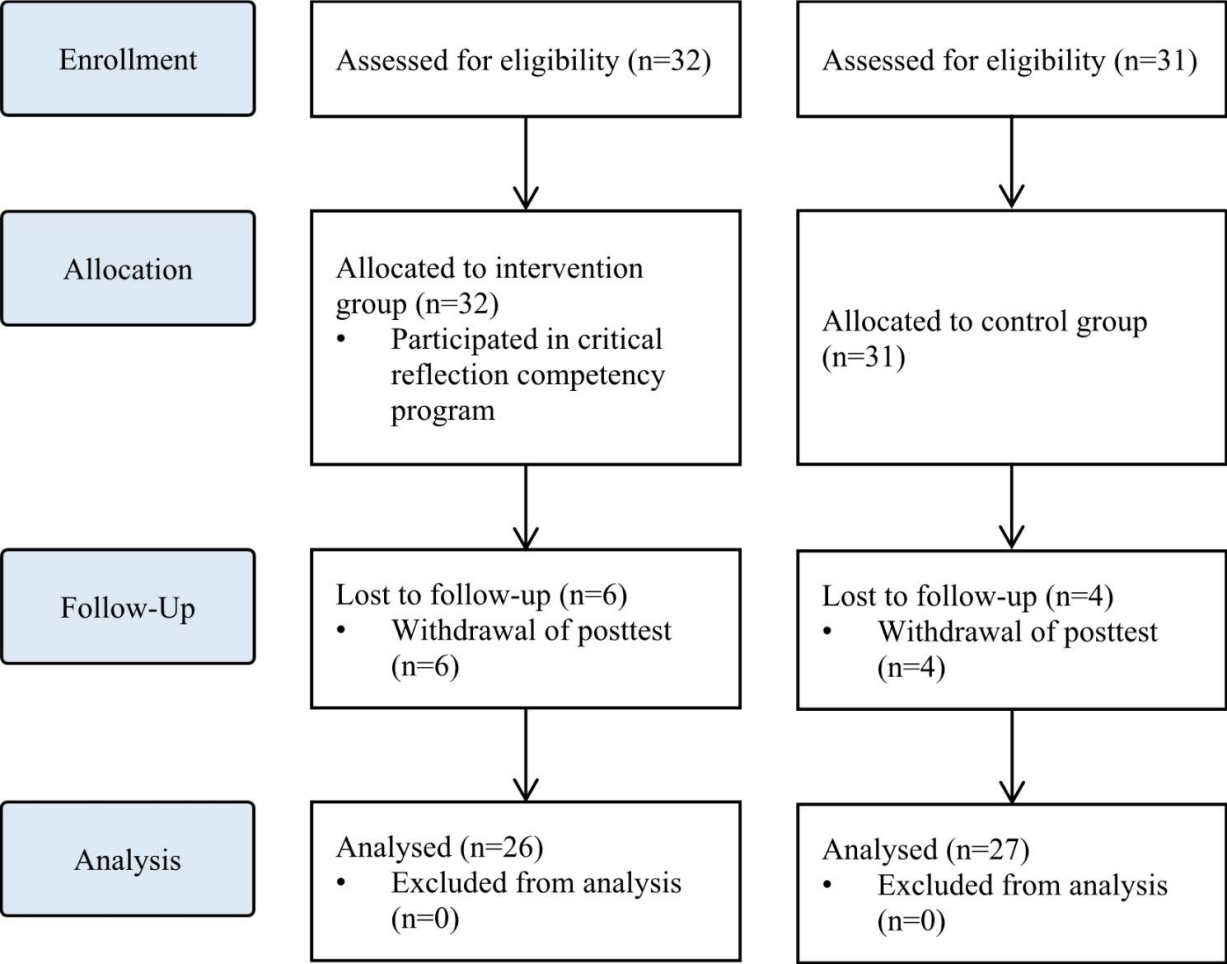
Flowchart of the study

The sample size required for the analysis was calculated using the G* Power 3.1.9.4. program with an effect size of 0.80, a significance level of 0.05, and a power of 0.80, following the literature [20]. By applying a self-reflection program for intensive care unit nurses [20], we calculated the effect size as large. Both the intervention and control groups required 26 participants. Considering a dropout rate of 20%, a total of 63 participants, including 32 in the intervention group and 31 in the control group, were recruited. From the intervention group, six participants who participated in the pre-test and completed all programs, but did not participate in the post-test, were excluded. In the control group, four participants who participated in the pre-test but not in the post-test were excluded. Thus, 26 and 27 participants in the intervention and control groups, respectively, were included in the final analysis [Fig. 2]. The pre-test for both groups was conducted in May 2021. Post-tests for the two groups were performed four weeks after the pre-test.

### Intervention

The intervention was developed and delivered by the first author, who has more than 15 years of teaching experience in nursing education, including critical reflection. The intervention was conducted between May 2021 and June 2021. Following previous studies that applied critical reflection in medical education [21, 22], the intervention was conducted once a week for 90 min, with a total of four sessions. Owing to COVID-19, real-time online sessions were used to minimize contact between participants working in medical institutions. Every week before the sessions, the contents of the session, schedule, and Uniform Resource Locator (URL) were sent to participants via e-mail.

**Fig. 3:**
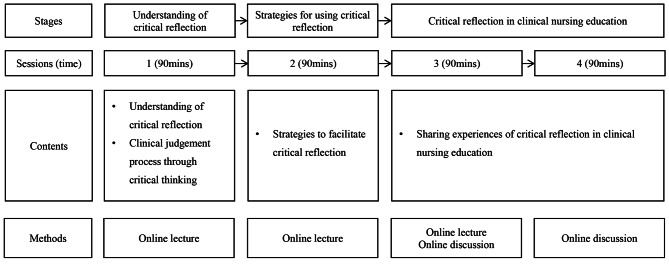
Critical reflection program for clinical nurse educators

The intervention consisted of the following three steps in four sessions: (1) understanding critical reflection, (2) strategies to use critical reflection, and (3) practical uses of critical reflection [Fig. 3]. Synchronous online lectures were conducted in the first and second sessions. The contents of the first session included understanding of critical reflection and the clinical judgment process through critical reflection. Based on the content of the first session, the second outlined educational strategies using critical reflection in nursing education and the direction of critical reflection. In the third and fourth sessions, clinical nurses with experience of critically reflecting on themselves were invited as guest speakers to share their experiences and facilitate online discussions. Online discussions were also conducted in real time, and feedback from guest speakers and the author was immediately provided.

### Measures

Online self-report surveys were conducted before and after the program to assess the program’s effects. In both pre- and post-tests, critical thinking disposition, nursing reflection competency, and teaching efficacy were assessed, as well as information about participants, such as gender, age, experience in nursing education, and the type of institution and the number of beds they affiliated with.

Critical thinking disposition was measured using Yoon’s Critical Thinking Disposition Scale [23]. This scale comprises 27 items: 5 on “intellectual eagerness/curiosity,” 4 on “prudence,” 4 on “self-confidence,” 3 on “systematicity,” 4 on “intellectual fairness,” 4 on “healthy skepticism,” and 3 on “objectivity.” The items were evaluated on a five-point Likert scale (one point for “strongly disagree” to five points for “strongly agree”); a higher score indicated greater critical thinking disposition. The scale has good reliability as evidenced by a Cronbach’s alpha of 0.84 at the time of the development versus the reliability of the scale in this study was Cronbach’s alpha of 0.78.

Nursing reflection competency was assessed using the Nursing-Reflection Questionnaire, developed by Lee et al. [24]. This scale comprises four factors with 15 items, including 6 items on “review and analysis nursing behavior,” 5 on “development-oriented deliberative engagement,” 2 on “objective self-awareness,” and 2 on “contemplation of behavioral change.” Each item was evaluated on a five-point Likert scale (one point for “strongly disagree” to five points for “strongly agree”), with a higher score indicating greater nursing reflection competency. The scale has good reliability as evidenced by a Cronbach’s alpha of 0.86 at the time of the development versus the reliability of the scale in this study was Cronbach’s alpha of 0.82.

Teaching efficacy was evaluated using the Teaching Efficacy Scale developed by Park and Suh [25] to evaluate clinical nursing instructors. This scale consisted of six sub-factors with 42 items, including 12 items on “student instruction,” 9 on “teaching improvement,” 7 on “application of teaching and learning,” 7 on “interpersonal relationship and communication,” 4 on “clinical judgment,” and 3 on “clinical skill instruction.” Each item was evaluated on a five-point Likert scale (one point for “strongly disagree” to five points for “strongly agree”), with a higher score indicating greater teaching efficacy. The scale has good reliability as evidenced by a Cronbach’s alpha of 0.97 at the time of the development versus the reliability of the scale in this study was Cronbach’s alpha of 0.93.

### Ethical considerations

This study was approved by the Institutional Review Board (IRB) of Ewha Womans University (IRB no. ewha-202105-0022-02). The need of written informed consent was exempted by IRB of Ewha Womans University. All methods were performed in accordance with the relevant guidelines and regulations. A description of the study, including its purpose, methods, and procedures, was posted on an online pre-test survey. Only those participants who agreed to participate were allowed to complete the questionnaire. The participants were also informed that they could withdraw from the study at any time and that the data of withdrawn participants would not be included in the final analysis. After the survey was completed, a mobile gift voucher was provided to those who agreed to provide their mobile phone number. Data were collected by researchers who did not participate in the program.

### Data analysis

The collected data were analyzed using SPSS for Windows (version 28.0). Non-normally distributed data were analyzed using non-parametric tests. Descriptive statistics were used to calculate the frequency, percentage, mean, and standard deviation of participants’ general and institutional characteristics. Chi-square, independent t-, and Mann-Whitney U tests were conducted to test the homogeneity of the general characteristics and pre-test scores. The Shapiro-Wilk test was conducted to test the normality of the data. To test the difference between the pre- and post-tests for each group, analysis of covariance (ANCOVA) was used. As there was a significant difference in age between the intervention and control groups, an ANCOVA with age as a covariate was conducted for the difference in changes in test scores between the pre- and post-test.

## Results

### Homogeneity test of general characteristics and dependent variables

All participants were female, with a mean nursing education experience of 27 and 23 months in the intervention and control groups, respectively. The homogeneity test of general and institutional characteristics, such as gender, nursing education experience, affiliated institution types, and the number of beds, were not statistically significant. However, the age differed significantly between the two groups. In the test for homogeneity of the pre-intervention scores, there were no significant differences in critical thinking disposition, nursing reflection competency, or teaching efficacy between the two groups, suggesting homogeneity of the dependent variables between the groups [Table 1].

**Table 1:** Homogeneity test on general characteristics and dependent variables (N = 53)

Variables	Exp. (n = 26)	Cont. (n = 27)	Χ^2^/t/Z	*p*
	n (%) or	n (%) or		
	Mean ± SD	Mean ± SD		
Gender				
Female	26 (100.0)	27 (100.0)		
Age (years)^*^	35.85 ± 5.19	30.30 ± 3.02	**5.711**	0.021
Experience with nursing educational work (month)^*^	27.81 ± 27.44	23.56 ± 21.29	0.632	0.808
Hospital type^**^				
Tertiary general hospital	16 (61.5)	6 (22.2)	4.875	0.087
Secondary hospital	10 (38.5)	21 (77.8)		
Number of hospital beds^*^	925.50 ± 452.13	786.41 ± 431.08	2.541	0.117
Critical thinking disposition^*^	3.61 ± 0.26	3.76 ± 0.21	-1.285	0.904
Nursing-reflection^***^	57.00 ± 3.42	59.63 ± 5.24	-2.198	0.139
Teaching efficacy^*^	157.04 ± 10.60	161.59 ± 14.77	-2.155	0.132

^*^ Independent t-test; ^**^ Chi-square test; ^***^ Mann-Whitney U test; Exp.=experimental group; Cont.=control group

### Effects of critical reflection competency program

The effects of the critical reflection competency program are shown in Table 2.

**Table 2:** Effects of critical reflection competency program (N = 53)

Variables	Groups	Pretest	Posttest	Adjusted Posttest	F(*p)*
		Mean ± SD		LSM ± SE	
Critical thinking disposition	Exp.(n = 26)	3.61 ± 0.26	3.82 ± 0.23	3.87 ± 0.04	**14.751**(< 0.001)
	Cont.(n = 27)	3.76 ± 0.21	3.79 ± 0.28	3.77 ± 0.04	
Nursing-reflection*	Exp.(n = 26)	57.00 ± 3.42	61.08 ± 3.64	60.86 ± 0.95	2.674(0.108)
	Cont.(n = 27)	59.63 ± 5.24	60.04 ± 6.50	59.04 ± 0.89	
Teaching efficacy	Exp.(n = 26)	157.04 ± 10.60	171.23 ± 10.86	171.98 ± 2.54	**11.047**(< 0.001)
	Cont.(n = 27)	161.59 ± 14.77	161.89 ± 16.91	160.48 ± 2.46	

^*^ Ranked ANCOVA; Exp.=experimental group; Cont.=control group; LSM = least mean square

In the post-intervention phase, scores of critical thinking disposition, nursing reflection competency, and teaching efficacy all improved compared to the pre-intervention phase, and were higher in the experimental group than in the control group. The critical thinking disposition scores before and after the intervention were 3.61 ± 0.26 vs. 3.87 ± 0.04 in the intervention group and 3.76 ± 0.21 vs. 3.77 ± 0.04 in the control group, respectively. The nursing reflection competency scores before and after the intervention were 57.00 ± 3.42 vs. 60.86 ± 0.95 in the intervention group and 59.63 ± 5.24 vs. 59.04 ± 0.89 in the control group. The teaching efficacy scores before and after the intervention were 157.04 ± 10.60 vs. 171.98 ± 2.54 in the intervention group and 161.59 ± 14.77 vs. 160.48 ± 2.46 in the control group.

Age, which was significantly different between the intervention and control groups, was treated as a covariate to conduct the ANCOVA. The changes in critical thinking disposition (F = 14.751, *p* < 0.001) and teaching efficacy (F = 11.047, *p* < 0.001) scores were significantly different between the two groups. However, there was no significant difference in the change in the nursing reflection competency (F = 2.674, *p* = 0.108) score between the two groups.

## Discussion

Reflective practice is crucial to clinical nurses’ professionalism. Reflective practice enables positive learning experiences through deep and meaningful learning, and is essential for integrating theory and practice. It also enables nurses to implement what they have learned into practice, understand their expertise, and develop clinical competencies [26]. In this respect, it is important for clinical nursing educators to have critical reflection competencies and promote experiential learning among new nurses. In this study, a critical reflection competency program was developed to enhance clinical nurse educators’ critical thinking and teaching competency.

This program was effective in improving critical thinking disposition. In interventions for critical reflection, various aspects, including the introduction of critical reflection and guidelines to promote critical reflection, such as small group discussions and feedback, can be considered [27]. The program reflected these aspects and helped improve participants’ critical thinking disposition. In the third and fourth sessions, synchronous discussions on sharing experiences of critical reflection were effective. This is consistent with previous studies in which discussions improved reflective competencies [21, 28]. Therefore, sharing experiences in the discussion section should be a key element of future educational interventions for critical reflection competency.

Furthermore, the program was effective in improving teaching efficacy. Teaching efficacy is the instructor’s belief in one’s own ability to organize and implement teaching [29], and is closely related to age, clinical experience, educational experience, professional development, and teaching competency [30–32]. Nurses who are more clearly aware of their roles as instructors tend to exhibit higher confidence in their teaching abilities [33, 34]. That is, the participants in this study were clearly aware of their roles and developed confidence by sharing their educational experiences about critical reflection.

However, the program did not have a significant effect on nursing reflection competencies. In a previous study [10], reflective practitioners (RPs) received four weeks of critical reflection training and trained new nurses for six months. During training, new nurses wrote critical reflective journals and RPs provided feedback and shared their experiences. In this study, it seems that the methods and frequency of using critical reflection in nursing education varied for each participant, resulting in insignificant results for nursing reflection competency. It is necessary to provide educational materials or guidelines so that nurse educators can use critical reflection in nursing education.

In this pilot study, the program was found to be effective in improving critical thinking disposition and teaching efficacy. The results show that the program can enhance the critical thinking disposition of nurse educators and help them develop teaching competency by critically reflecting on their educational experiences as instructors [35, 36]. Therefore, various educational programs and training systems related to critical reflection are required [37]. However, many medical institutions find it difficult to provide sufficient educational support to nurses because of limited costs, time, and physical space [38]. Online real-time lectures and case-based discussions of the developed program can be useful alternatives to overcome barriers to nursing education support. Additionally, more effective educational content and platforms using e-learning can be developed based on the results of this study.

In this study, the critical reflection competency program for clinical nurse educators was developed and conducted. The program was an educational intervention to improve the critical reflection competency of clinical nurse educators in real time online. Several limitations must be considered when interpreting the present findings. The developed program did not affect nursing reflection competencies. Further, the post-test was conducted shortly after program completion. Therefore, there may be limitations to evaluating whether the developed program improves the quality of nursing education. In addition, the participants in this study were allocated regardless of their hospital’s characteristics. Considering variables such as the size of the hospitals, the number of new nurses, and the number of participants per hospital, it is necessary to assign nurse educators the intervention and control groups and to verify the effects of the program. Future studies should consider improving the study design to measure the long-term effects of the program and randomize the participants.

## Conclusion

The effects of the program on critical thinking disposition, nursing reflection competency, and teaching efficacy were assessed. The results showed that the program was effective in improving the critical thinking disposition and teaching efficacy of nurse educators. However, there was no significant difference in nursing reflection competency, but it may vary depending on the methods or time of using critical reflection in nursing education. Therefore, it is necessary to provide the critical reflection utilization strategies that can be used by clinical nurse educators in the clinical settings. In addition, further research, such as evaluating the reflective practice of new nurses trained by clinical nurse educators, is needed. This suggests that the critical reflection competency program should be expanded in the future for nurse educators. It is necessary to develop e-learning content and educational platforms to expand the program, and it should be possible to share the experience of critical reflection in various forms. Also, sufficient support for competency improvement of nurse educators is needed to effectively use critical reflection in nursing education. Nursing leaders in hospital and healthcare settings should recognize the importance of using critical reflection in clinical practice and improving the competency of clinical nursing educators who educate new nurses, and make efforts to improve the quality of nursing education through support for these. Lastly, based on the results of this study, we recommend further longitudinal and randomized studies to evaluate additional effects of the program.

## Data Availability

The datasets used or analyzed during the current study are available from the corresponding author on reasonable request.
